# Immunosuppression in Gliomas *via* PD-1/PD-L1 Axis and Adenosine Pathway

**DOI:** 10.3389/fonc.2020.617385

**Published:** 2021-02-15

**Authors:** Thamiris Becker Scheffel, Nathália Grave, Pedro Vargas, Fernando Mendonça Diz, Liliana Rockenbach, Fernanda Bueno Morrone

**Affiliations:** ^1^ Laboratório de Farmacologia Aplicada, Escola de Ciências da Saúde e da Vida, Pontifícia Universidade Católica do Rio Grande do Sul (PUCRS), Porto Alegre, Brazil; ^2^ Programa de Pós-Graduação em Biologia Celular e Molecular, Escola de Ciências da Saúde e da Vida, Pontifícia Universidade Católica do Rio Grande do Sul (PUCRS), Porto Alegre, Brazil; ^3^ Programa de Pós-Graduação em Medicina e Ciências da Saúde, Escola de Medicina, Pontifícia Universidade Católica do Rio Grande do Sul (PUCRS), Porto Alegre, Brazil

**Keywords:** glioma, immunosuppression, adenosine, PD-1/PD-L1, tumor microenvironment

## Abstract

Glioblastoma is the most malignant and lethal subtype of glioma. Despite progress in therapeutic approaches, issues with the tumor immune landscape persist. Multiple immunosuppression pathways coexist in the tumor microenvironment, which can determine tumor progression and therapy outcomes. Research in immune checkpoints, such as the PD-1/PD-L1 axis, has renewed the interest in immune-based cancer therapies due to their ability to prevent immunosuppression against tumors. However, PD-1/PD-L1 blockage is not completely effective, as some patients remain unresponsive to such treatment. The production of adenosine is a major obstacle for the efficacy of immune therapies and is a key source of innate or adaptive resistance. In general, adenosine promotes the pro-tumor immune response, dictates the profile of suppressive immune cells, modulates the release of anti-inflammatory cytokines, and induces the expression of alternative immune checkpoint molecules, such as PD-1, thus maintaining a loop of immunosuppression. In this context, this review aims to depict the complexity of the immunosuppression in glioma microenvironment. We primarily consider the PD-1/PD-L1 axis and adenosine pathway, which may be critical points of resistance and potential targets for tumor treatment strategies.

## Introduction

Cancer is characterized by genetic instability and heterogeneity in the tumor microenvironment (TME). Currently, one of the major challenges in cancer treatment is to block the multifaceted network of tumor mechanisms that cause immunosuppression and resistance to cell death (1, 2).

Gliomas are the most aggressive primary brain tumors in adults, and are of different genetic, phenotypic, and pathological subtypes, depending on the glial lineage from which they arise (3). Glioblastoma multiforme (GBM) is the most malignant subtype of diffuse glioma, and remains the most lethal among brain tumors (3, 4). Similar to other malignances, genetic and phenotypic variability within GBM present problems for the treatment of these tumors (5, 6).

Despite advances in modern medicine, the prognosis for malignant glioma patients remains just over a year. Therefore, several avenues, such as tumor resistance, need to be explored to improve therapeutic approaches (7, 8). Tumor resistance is related to redundant and synergic immunosuppressive pathways coexisting in the TME. Malignant and host cells create a specific niche, where cellular interactions shape the profile of cytokines and chemokines, favoring pro-tumoral activities (9).

Recent evidence has shown that tumors are proficient at evading immunostimulatory responses and resisting standard therapy by producing adenosine (ADO) and upregulating molecules like programmed cell death 1 (PD-1) that function as immune checkpoints (9, 10). Therefore, this review aims to depict the complexity of the immune system in the glioma microenvironment, including the role of the PD-1/PD-L1 axis and adenosine pathway in the maintenance of immunosuppression and resistance to glioma treatments.

## Immune System in Gliomas

### Tumor-Associated Immunosuppression

The TME has been described as a regulator of tumor progression as well as a mediator of successful therapy. The complexity of tumor niche is shaped by a variable combination of stromal cells, endothelial cells, fibroblasts, cancer stem cells and immune system. Specially, stromal and cancer stem cells have been described by a significant involvement on glioma initiation, maintenance, and progression. In fact, cancer stem cells can suppress cytotoxic responses and modulate immune and endothelial cell functions, suggesting an important role of these cells on immunosuppressive tumor site (11, 12). Importantly, studies have demonstrated an increasing significance of the immune infiltrate and its products in the process of tumor malignancy (10, 13, 14). In GBM, resident microglia and macrophages represent up to one-third of the tumor mass and may have pro-tumorigenic functions (15).

Microglial cells are considered “plastic” due to their ability to change their functions based on environment. These cells may exhibit pro-inflammatory (M1) or immunosuppressive (M2) functions (15–17). All macrophages produce several cytokines such as tumor necrosis factor (TNF) and interleukins (IL-1, IL-6, IL-8, and IL-12), which influence the generation of effector cells and activation of lymphocytes (14). Previous data has shown that the interaction between glioma and microglia is very complex and may not be beneficial for tumor resolution. Indeed, microglia cells co-cultured with glioma cells lack phagocytic ability against tumor cells (16).

Immunosuppression in gliomas involves dynamic crosstalk between tumor and stromal cells, tumor-associated macrophages (TAMs), microglia, regulatory T cells (Tregs), and tumor-infiltrating lymphocytes (TIL) (17–19). Generally, the number of CD4^+^ lymphocytes is lower than that of CD8^+^ lymphocytes in a GBM environment, but it has been observed that the numbers of both CD4^+^ and CD8^+^ cells increase with tumor grade (20). Despite the presence of these lymphocytes in the GBM microenvironment, effector T cells do not function properly and M2 macrophages are unable to promote CD4^+^ and CD8^+^ polarized immune responses, which are important for the regulation of Tregs (21).

The release of chemokines such as C-C motif chemokine ligand 2 (CCL2) is critical for the recruitment of Tregs and myeloid derived suppressor cells (MDSCs). MDSCs alter the TME and suppress immune responses by blocking CD8^+^ cells and inhibiting the function of natural killer cells (NK) (9, 19, 22). NK cells express death receptor ligands, which can induce caspase-dependent apoptosis in target cells, and can thus kill cancer cells (23). This cytotoxic action is limited by GBM-HLA-G expression, which protects tumors from T cells and NK-mediated killing. Moreover, NK cells are reduced in GBM patients (23, 24).

Studies have shown influences of effector and regulator T cells on the prognosis of cancer patients. For example, Tregs play a significant role in the immune response in the TME since they mediate immunotolerance by suppressing the function of effector T cells (9, 23). GBM patients showed an increased proportion of Tregs among CD4^+^ cells, contributing to the reduced immune response (25). In addition, the removal of the Treg fraction from patients with GBM rescues T cell proliferation and pro-inflammatory cytokine production to standard levels. This reveals the critical role of Tregs in glioma-mediated immunosuppression (26).

### The PD-1/PD-L1 Axis

Interest in immune-based treatments of cancer has been renewed after the discovery of immune checkpoint inhibitors. Recently, the co-Nobel Prize in Physiology or Medicine was awarded to Tasuko Honjo, who showed the negative regulation of T cells mediated by the PD-1 pathway (27, 28). Thus, the expression and activity of immunological checkpoints have emerged as the main immunosuppressive mechanisms in gliomas (29, 30).

The transmembrane co-receptor PD-1 (CD279), encoded by the PDCD1 gene, belongs to the family of immunoglobulins and is expressed predominantly by activated T lymphocytes (31). PD-1 is often activated by PD-L1 (B7-H1; CD274), one of the ligands known to be expressed by antigen presenting cells (APCs), B lymphocytes, and parenchymal cells. PD-L2 (B7-DC; CD273) is another ligand for PD-1 and is expressed by fewer cells than PD-L1 (31–33). In normal conditions, PD-1/PD-L engagement occurs controlling a prolonged activation of immune system, often avoiding autoimmunity processes. It is known that PD-1 interaction provides T-cell inhibitory signals. PD-1/PD-L engagement during TCR stimulation leads to tyrosine phosphorylation of the PD-1 cytoplasmic tail on high affinity sites for SH2 domain-containing phosphatase (SHP-2 and SHP-1), resulting in the dephosphorylation of proximal signaling molecules which decrease T cell proliferation and survival by attenuate PI3K and Akt pathways (31, 34).

Importantly, expression of PD-L1 has been detected in glioma (35–37). Moreover, PD-L1 expression in tumor cells is related to levels of malignancy, and high PD-L1 expression is associated with greater invasiveness and aggressiveness of GBM cells (38, 39). Studies have shown heterogeneity of PD-L1 expression in tumor mass such that greater expression is seen at the edges of the tumor than in the core. This could also facilitate immune evasion and invasiveness of gliomas (38, 40).

The expression of PD-L1 in the TME is regulated mainly by cytokine and receptor antigen signaling (31). Interferon gamma (IFN-γ) is the major PD-L1 regulation factor in tumor cells and reflects ongoing antitumor immune activity. In addition, oncogenic mutations, such as loss of phosphatase and tensin homolog (PTEN) in glioma, can activate PD-L1 expression in tumor cells (31, 41, 42).

The PD-1/PD-L1 pathway has been appropriated by tumor cells to resist antitumor responses and facilitate tumor survival (42, 43). Influenced by hypoxia, cytokines, and oncogenes, GBM cells express PD-L1, which engages with the PD-1 receptor primarily on T cells and attenuates its functions, effectively reducing the antitumor activity of these cells (42).

A subset of lymphocytes (Tregs) has emerged as a critical target in cancer therapy. Tregs express both PD-1 and PD-L1, and the generation, immunosuppression, and interaction of Tregs with effector T cells could be, at least in part, modulated by PD-1/PD-L1 binding (44, 45). Francisco et al. have shown that PD-L1 can induce and maintain the expression of FOXP3 in induced Tregs, suggesting that PD-L1 may control Treg plasticity (46).

GBM cells were also able to upregulate PD-L1 expression in tumor-infiltrating macrophages *via* modulation of IL-10 signaling (29). Macrophages may express PD-1 and PD-L1 (47). PD-1 positive TAMs exhibit decreased phagocytic potential and PD-1 blockade improves macrophage functionalities, besides reducing tumor growth in mouse models of cancer (48).

The use of PD-1 inhibitors is becoming an effective strategy for the treatment of cancer, and several preclinical and clinical studies have been conducted for GBM (30, 49). In fact, immune checkpoint inhibitors may reverse the immunosuppressive condition and restore dysfunctional or “exhausted” T cell function in cancer (39). However, some patients remain unresponsive to PD-1/PD-L1 blockade. Therefore, fresh clinical trials to evaluate tumor resistance in PD-1/PD-L1 immunotherapy in GBM patients are required (39, 50).

### Immunomodulation by Adenosine Pathway in Gliomas

Adenosine 5′-triphosphate (ATP) is the main energy molecule produced by cellular respiration. It has multiple release routes and is involved in practically all cellular responses (51). It is known that during cancer growth and progression, ATP and its main metabolite, ADO, are actively secreted or generated in the extracellular space, and accumulate to high levels in the TME (52–54).

Physiologically, extracellular ATP (eATP) functions as a “danger” signal alerting the immune system to the presence of inflammation, and is crucial for inflammasome activation and the concomitant release of cytokines (54, 55). These effects are mediated *via* P2 receptors, which are subdivided into two subfamilies: P2X ionotropic ion channel receptors (P2X1-7) and P2Y G-protein-coupled receptors (P2Y_1, 2, 4, 6, 11, 12, 13, 14_) (53–55). These purinergic receptors display distinct agonist affinity and specificity, affecting both tumor and immune cells, depending on the eATP levels available in the TME (56). Different innate and adaptive immune responses are generated through activation of P2 receptors by eATP (**Table 1**). Particularly, the participation of P2X7 in inflammation is extensive, and has been better characterized compared to that of other P2 receptors (54, 55, 71–74). The direct role of P2X7 in carcinogenesis is still controversial, but it is known that cell growth or death is triggered according to the cell type that expresses P2X7 and their activation level (75).

**Table 1:** Functional immune responses triggered by nucleotides and nucleosides actions in glioblastoma microenvironment.

		Main purinergic receptors	Immune outcome	Immune cell profile	Cytokine & chemokine profile	Ref
Immunostimulatory	ATP	P2X1	**Proinflammatory response**	Chemotaxis of neutrophils; chemotaxis and phagocytosis of macrophages; release of chemokines and cytokines from eosinophils; T cell activation.	IL-2, IL-8, IL-12, TNF-α (increased)	(57–61)
		P2X4	**Proinflammatory response**	Microglia activation and proliferation; macrophages stimulation and maturation; stimulation of dendritic cells; T cell activation.	IL-2, IL-12, TNF-α (increased)	(54, 57, 59–62)
		P2X5	**Adaptative immune response**	T and B lymphocytes activation.	IL-2 (increased)	(57, 60)
		P2X7	**Proinflammatory response** **NLRP3 inflammasome activation**	Recruitment of macrophages and neutrophils; inhibition of the suppressive potential of Tregs.	IL-1β, IL-12, IL-18, IFN-γ, TNF-α, CCL-3, CXCL2 (increased)	(54, 57, 60–65)
		P2Y_2_	**Innate immune response**	Chemotaxis of eosinophils, monocytes/macrophages, microglia, and dendritic cells; degranulation of neutrophils.	MCP-1, CCL2, IL-6, IL-8, IL-33 (increased)	(57, 66)
Immunosuppressive	ADO	A_2a_	**Immunosuppressive response**	Macrophage differentiation into M2 phenotype; T cell anergy; increase differentiation and suppressive effect of Treg; upregulation of immune checkpoint receptors (e.g., PD-1, CTLA-4); reduction of NK cell cytotoxicity; inhibition of neutrophil and microglial chemotaxis; modulation of chemokines profile in neutrophils.	IL-10, VEGF, TGF-β (increased) IL-12, TNF-α, nitric oxide; IFN-γ (decreased)	(57, 64, 67–70)
		A_2b_	**Anti-inflammatory response**	Macrophage differentiation into M2 phenotype; reduction of monocyte differentiation to dendritic cell; MDSCs expansion; reduction of adherence and degranulation of neutrophils.	Arginase 1, IL-10, VEGF, IL-6; TGF-β (increased) IL-12, TNF-α (decreased)	(57, 68, 70)

ATP, adenosine 5′-triphosphate; ADO, adenosine; CCL3, C-C motif chemokine ligand 3; CXCL2, C-X-C motif ligand 2; CTLA-4, cytotoxic T-lymphocyte antigen 4; IFN-γ, interferon γ; IL-, interleukine; MCP-1, monocyte chemoattractant protein-1; MDSC, myeloid-derived suppressor cell; NK, natural killer; NLRP3, sensor molecule (NOD- LRR- and pyrin domain-containing protein 3); PD-1, programmed cell death 1; TGF-β, transforming growth factor beta; TNF-α, tumor necrosis factor alpha; VEGF, vascular endothelial growth factor.

P2 receptors are assumed to be inactive in normal physiological conditions, where ATP-dependent signaling should be at baseline levels. Ectoenzymes, such as NTPDase1 (CD39) and ecto-5′-nucleotidase (CD73), maintain levels of extracellular ATP, which is crucial to avoid P2 receptor desensitization (76).

In the TME, eATP is quickly hydrolyzed to AMP by CD39 of TILs which is then efficiently converted to the immunosuppressant ADO by CD73 expressed in glioma cells (77). ATP hydrolysis drives the immune response to collaborate with tumor growth, making the CD39/CD73 axis an important regulator of immune effector function. This is a hallmark of cancer (78–81). Interestingly, CD39 inhibition can restore TIL function, and a single nucleotide polymorphism has been identified that may predict dysfunctional CD39^+^ expression in TILs in some solid tumors (81).

The suppressive role of ADO in the TME is primarily mediated by cytotoxicity, anti-inflammatory cytokine production, and restriction of immune cell infiltration (79). Adenosine effects are mediated by P1 receptors (A_1_, A_2a_, A_2b_, and A_3_). Interestingly, the pro-tumoral effects of ADO occur mainly through A2 receptors, as depicted in **Table 1**. Physiologically, ADO orchestrates tissue recovery after initial inflammation, which involves the decrease of M1 phenotype, cell proliferation, and angiogenesis. This sets the stage for tumor growth. Hence, the ADO signaling pathway may be an important therapeutic target (79, 80, 82, 83).

## The PD-1/PD-L1 Axis and Adenosine Pathway in Gliomas

Typically, tumor growth involves disruption of the surrounding microenvironment, in which extracellular nucleotides might confer immunomodulatory properties that are critical for driving glioma immune escape. One of the main mechanisms of tumor immune evasion is the generation of high levels of ADO mediated by excessive activity of ectonucleotidases (83–85).

An effective immunosuppressive environment is maintained when the actions of ADO are synergistic or additive to other immunosuppressive mechanisms. There is growing evidence that immunosuppressive proteins, such as PD-1 and PD-L1, can be increased in the TME by the same mechanism that is implicated in hypoxia-mediated adenosinergic immunosuppression (86). Extracellular ADO increases in hypoxic conditions, concomitant with upregulation of CD39 and CD73. In addition, the oxygen deprivation in the tumor core is related to the upregulation of immunoregulatory mechanisms such as PD-L1 expression in glioma cells, making them resistant to T cell-dependent cytotoxicity (87).

Notably, it was suggested that ADO also induces increase in PD-1 levels (88) because ADO signaling may positively regulate TGF-β levels. TGF-β is mainly involved in stopping effector T cell activation and stimulating the activity of antigen presenting cells that express PD-1 (89). In the presence of TGF-β, CD4^+^ cell activation may predominantly generate inducible Tregs (90). These cells primarily express CD39, while GBM cells express high levels of CD73, suggesting that cancer and immune cells can cooperate to promote local adenosinergic immunosuppression. Accordingly, a vicious cycle is formed, favoring the upregulation of the PD-1/PD-L1 axis that maintains a complex synergism between the ADO pathway and immune checkpoint axis (77, 91, 92).

Additionally, ADO is involved in macrophage activation, predominantly *via* A_2a_ (A_2a_R) and A_2b_ receptors (A_2b_R). A_2b_R stimulation during macrophage differentiation could skew macrophages toward the M2 phenotype. M2 macrophages can express immunoregulatory molecules such as arginase, TGF-β, and PD-1/PD-L1 proteins, resulting in the downregulation of cellular immune responses (93).

**Figure 1:**
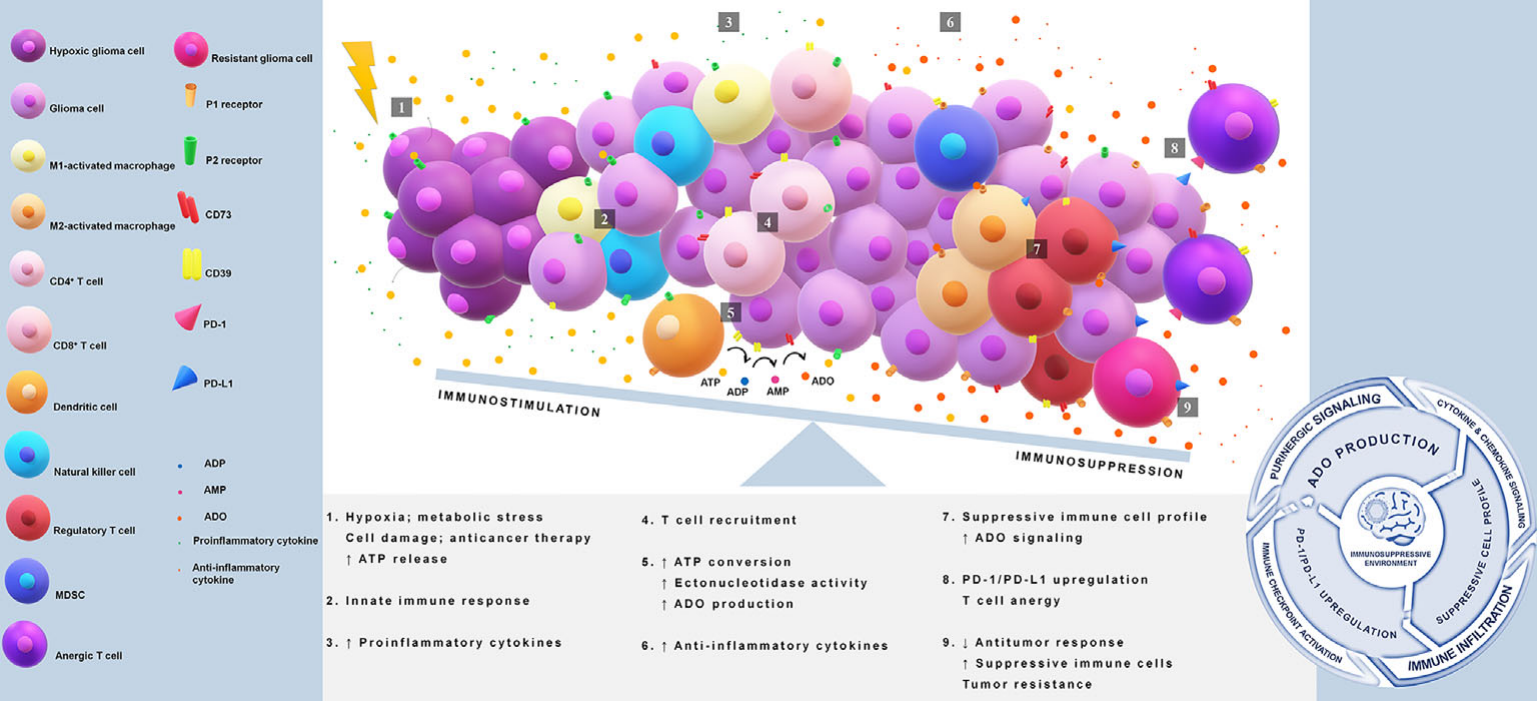
Immunosuppression in glioblastoma *via* PD-1/PD-L1 axis and adenosine pathway. Tumor core acquires reduction in the oxygen supply causing a release of high amounts of ATP. This nucleotide acts as a damage-associated molecular pattern (DAMP) and starts immune activation. Extracellular ATP binds to P2 receptors and triggers proinflammatory responses through the induction of cytokines and chemokines. A disbalance in the ATP concentration gradient leads to an upregulation of CD39/CD73 axis, favoring adenosine production. Adenosine is a key molecule that initiates a suppressive immune cell infiltration and drives the activation of PD-1/PD-L1 axis. The immunosuppressive loop is maintained indirectly by ATP release and adenosine signaling, which avoids antitumor defenses, promotes immunosuppressive cell profile, and induces upregulation of immune checkpoints. ATP, adenosine 5′-triphosphate; ADO, adenosine; CD39 or ectonucleoside triphosphate diphosphohydrolase 1, cluster of differentiation 39; CD73 or ecto-5′-nucleotidase, cluster of differentiation 73; DAMP, damage-associated molecular pattern; MDSC, myeloid-derived suppressor cells; PD-1, programmed cell death 1; PD-L1, programmed cell death ligand 1.

Overall, the multifaceted role of ADO in tumor immune evasion is seen in its promotion of pro-tumor rather than antitumor immune responses, dictation of Treg function, inhibition of effector T cells, modulation of anti-inflammatory cytokines, and induction of immune checkpoints as illustrated in **Figure 1** (83, 84, 86, 88, 89, 94).

Taken together, the ADO pathway and the PD-1/PD-L1 axis may act synergistically to modify the TME, favoring tumor progression. Based on this landscape, the GBM standard treatment should be multimodal, involving maximal surgical removal followed by radiotherapy (RT) and/or temozolomide (TMZ). Despite such treatments, refractoriness is often observed (95, 96).

TMZ and RT have several immune modulatory effects on the TME. In addition to immune activation, RT and TMZ therapy may even worsen the immunosuppressive system in GBM. This is because both interventions induce immunogenic cell death, and consequently release immunogenic factors such as ATP (97, 98). ATP binding to P2X7 purinergic receptor is a signal that primes the immune system against tumor (99). However, glioma therapy also increases the expression of CD39/CD73. Hence, it is possible that ADO rapidly rises in the TME. RT also stimulates TGF-β and chemokines that promote the recruitment of immunosuppressive cells; therefore, the activity of the PD-1/PD-L1 axis increases. In addition to Tregs recruitment, RT-induced ATP release also can be related to Treg differentiation from naïve CD4^+^ cell *via* A_2b_R (100, 101).

Interestingly, some studies have shown irradiation-induced PD-L1 expression through an IFN-dependent pathway (102). Xia et al. (90) showed that under RT, PD-L1 expression in GBM cells is greater than that observed without radiation, and that the inhibition of PD-L1 increased radio-sensitivity in these cells (90). High PD-L1 expression was also associated with high numbers of M2 macrophages and Tregs, and low CD8^+^ cells in the TME, favoring high levels of ADO. Consequently, the immunosuppressive TME resulting from PD-L1-induction could be an important mechanism of tumor radio-resistance (103).

## PD-1/PD-L1 Axis Blockade and Purinergic Modulation Therapy

Recently, there has been a surge in the research and development of immunotherapies in cancer, using the PD-1/PD-L1 axis blockade as a strategy to reduce tumor immune evasion (103). Anti-PD-1 immunotherapy has been shown to be successful in prolonging responses in only a fraction of patients (36, 47). There is a subset of them who fail to overcome the immunosuppression, even they can mount an antitumor response. Consequently, the focus of research has changed toward uncovering intrinsic factors that contribute to treatment failures. Currently, the ADO pathway is considered a barrier for the efficacy of immunotherapies (103, 104).

As seen in some solid tumors, alternative immunomodulatory molecules, including CD39, CD73 and A_2a_R, are upregulated in response to anti-PD-1 monoclonal antibody (mAb) (88, 104). Beavis et al. showed that CD73^+^ tumor cells restrict anti-PD-1 efficacy, and that this effect was relieved by concomitant treatment with an A_2a_R antagonist (104). Li et al. demonstrated that CD39 inhibition sensitizes tumor-resistant models to anti-PD1, and that blocking CD39 activity is associated with the enrichment of cytotoxic T cells in the TME and upregulation of inflammatory markers on these infiltrates (105).

Various clinical trials that evaluate purinergic modulation therapy along with anti-PD-1 mAb or anti-PD-L1 mAb are currently active or in the recruitment phase for multiple cancer types (**Supplementary Table S1**). In fact, simultaneous therapy using PD-1 inhibitors and targeting the adenosine pathway was more effective in improving survival, reducing tumor growth, and limiting metastasis than single therapy in some types of cancer (106–108). Furthermore, there is a rising range of anti-CD73 mAbs being tested in combination with other immunotherapies, generating encouraging results (100, 101, 109).

GBM is one of the most immunologically “cold” tumors among all cancers. The PD1/PD-L1 target characterizes a potential strategy for conversion of the “cold” GBM microenvironment into a “hot” microenvironment to enhance the immune response to antitumor immunotherapy (110). Therefore, anti-PD-1/PD-L1 is an emerging therapeutic possibility in gliomas (111). Since PD-1/PD-L1 blockades do not significantly promote global survivor in patients with recurrent GBM compared with standard therapy, clinical trials are exploring association between anti-PD-1/PD-L1 mAb with standard radio/chemotherapy and bevacizumab or new therapies such as genetically engineered T cells and vaccines (39, 111, 112). Most studies are undergoing clinical trials evaluation and the results still have not provided decisive conclusions (**Supplementary Table S2**).

Overall, the study of alterations in “purinoma” caused by immune checkpoint inhibitors would likely provide insights for the development of interventions to overcome the immunosuppressive glioma environment and boost immune responses generated by immunotherapies.

## Conclusion

The environment surrounding tumors directly impacts their progression. Multiple redundant and compensatory pro-tumor pathways coexist in the TME and are closely related to the success of therapeutic treatments. Immune checkpoint inhibitors help in cancer treatment, though it is not effective in some patients. Thus, immunosuppression remains a major obstacle to therapeutic success. Studies on the relationship between purinergic signaling and inflammation show that the ADO pathway and PD-1/PD-L1 axis have a close relationship and act together to create a favorable environment for tumor immune evasion. The eATP-adenosine axis has a specific role in pro-tumor immune responses including upregulating the PD-1/PD-L1 axis. The ADO pathway has been identified as the main compensatory route involved in the maintenance of immunosuppression in patients using anti-PD-1 immunotherapy, through a drop in innate or adaptive immunity. Therefore, future research should focus on concomitant disruption of the ADO pathway and PD-1/PD-L1 axis to avoid cancer resistance.

## Author Contributions

TS, NG, PV, FD, and LR critically appraised the literature and wrote. FM reviewed and approved final version of the manuscript.

## Funding

The authors thank Coordenação de Aperfeiçoamento de Pessoal de Nivel Superior–Brasil [CAPES, Finance Code 001] and Conselho Nacional de Desenvolvimento Científico e Tecnológico [CNPq, project no. 310317/2018-5] for financial support.

## Conflict of Interest

The authors declare that the research was conducted in the absence of any commercial or financial relationships that could be construed as a potential conflict of interest.
