# Supplementary material for: Immunosuppression in Gliomas via PD-1/PD-L1 Axis and Adenosine Pathway
Source: Front Oncol. 2021 Feb 15;10:617385. doi: 10.3389/fonc.2020.617385 (PMC7919594; doi:10.3389/fonc.2020.617385)
Supplement: Supplementary file 1 [file Table_1.docx]

**Table S1. Summary of current clinical trials for anti-PD-1/PD-L1 mAb along with the adenosine pathway target in cancer.**

| Agent | Target | Purinergic combination target | Clinical trial identifier | Phase | Status |
| --- | --- | --- | --- | --- | --- |
| AK104 Bispecific | PD-1/CTLA-4 | Anti-CD73 mAb | NCT04572152 | Phase I trial in patients with advanced or metastatic solid tumors. | Not yet recruiting. |
| Pembrolizumab | PD-1 | Anti-CD73 mAb  Anti- A2AR mAb | NCT03454451 | Phase I in multiple cancer. | Recruiting. |
| Pembrolizumab | PD-1 | Anti-CD73 mAb | NCT04148937 | Phase I in patients with advanced cancer. | Recruiting. |
| Pembrolizumab | PD-1 | Anti-CD39 mAb | NCT03884556 | Phase I in patients with lymphoma or solid tumor malignancies. | Recruiting. |
| PDR001 | PD-1 | Anti-CD73 mAb  Anti- A2AR mAb | NCT03549000 | Phase I in multiple cancer. | Recruiting. |
| PDR001 | PD-1 | Anti- A2AR mAb | NCT02403193 | Phase I/II in patients with non-small cell lung cancer. | Active, not recruiting. |
| PDR001 | PD-1 | Anti- A2AR mAb | NCT03207867 | Phase II in patients with selected advanced solid tumors and non-hodgkin lymphoma. | Recruiting. |
| Budigalimab | PD-1 | Anti-CD39 mAb | NCT04336098 | Phase I in patients with advanced solid tumors. | Recruiting. |
| Zimberelimab | PD-1 | Anti-A2A/A2BR mAb | NCT03629756 | Phase I in patients with advanced malignancies. | Active, not recruiting. |
| Zimberelimab | PD-1 | Anti-A2A/A2BR mAb | NCT04262856 | Phase II in patients with non-small cell lung cancer. | Recruiting. |
| Zimberelimab | PD-1 | Anti-CD73 mAb  Anti-A2A/A2BR mAb | NCT04381832 | Phase I/II in patients with metastatic castrate resistant prostate cancer. | Recruiting. |
| Zimberelimab Pembrolizumab | PD-1 | Anti-A2A/A2BR mAb | NCT03846310 | Phase I in patients with lung cancer. | Recruiting. |
| Durvalumab | PD-L1 | Anti-CD73 mAb | NCT03616886 | Phase I/II in patients with triple negative breast cancer. | Recruiting. |
| Durvalumab | PD-L1 | Anti-CD73 mAb | NCT02503774 | Phase I in patients with select advanced solid tumors. | Active, not recruiting. |
| Durvalumab | PD-L1 | Anti-CD73 mAb | NCT03875573 | Phase II in patients with breast cancer. | Active, not recruiting. |
| Durvalumab  Tremelimumab | PD-L1  CTLA-4 | Anti-CD73 mAb | NCT03267589 | Phase II in patients with ovarian cancer. | Recruiting. |
| Atezolizumab | PD-L1 | Anti-CD73 mAb | NCT03835949 | Phase I in patients with advanced or metastatic cancer. | Recruiting. |
| Atezolizumab | PD-L1 | Anti- A2AR mAb | NCT02655822 | Phase I in patients with renal cell cancer and metastatic castration resistant prostate cancer. | Recruiting. |

Abbreviations: A2AR, adenosine receptor subtype; A2BR, adenosine receptor subtype; CD39 or ectonucleoside triphosphate diphosphohydrolase 1, cluster of differentiation 39; CD73 or ecto-5′-nucleotidase, cluster of differentiation 73; CTLA-4, cytotoxic T-lymphocyte antigen 4; PD-1, programmed cell death 1; PD-L1, programmed cell death ligand 1.

**Table S2. Current clinical trials for anti-PD-1/PD-L1 mAb in high grade glioma.**

| Agent | Target | Combination Agent | Clinical trial identifier | Phase | Status |
| --- | --- | --- | --- | --- | --- |
| Nivolumab | PD-1 | - | NCT03925246 | Phase II in patients with recurrent IDH mutated high-grade gliomas. | Active, not recruiting. |
| Nivolumab | PD-1 | DC vaccine | NCT02529072 | Phase I in patients with recurrent grade III and grade IV brain tumors. | Completed. |
| Nivolumab | PD-1 | Standard radiochemotherapy | NCT03576612 | Phase I in patients with newly diagnosed high-grade gliomas. | Recruiting. |
| Nivolumab | PD-1 | Anti-CSF1 receptor | NCT02526017 | Phase I in patients with selected advanced cancers. | Completed. |
| Nivolumab | PD-1 | - | NCT03718767 | Phase II in patients with IDH-mutated gliomas. | Recruiting. |
| Nivolumab | PD-1 | - | NCT03557359 | Phase II in patients with recurrent or progressive IDH mutant gliomas with prior exposure to alkylating agents. | Recruiting. |
| Pembrolizumab | PD-1 | Standard radiochemotherapy | NCT03899857 | Phase II in patients newly diagnosed glioblastoma. | Recruiting. |
| Pembrolizumab | PD-1 | DC vaccine | NCT04201873 | Phase I in patients with surgically accessible recurrent/progressive glioblastoma. | Recruiting. |
| Carilizumab | PD-1 | Apatinib, small-molecule tyrosine kinase inhibitor. | NCT04588987 | Phase II in patients with recurrent high-grade glioma. | Recruiting. |
| Camrelizumab | PD-1 | Standard radiochemotherapy | NCT04583020 | Phase II in patients with newly diagnosed glioblastoma. | Recruiting. |
| Retifanlimab | PD-1 | Stereotactic Radiosurgery and Anti-GITR agonist | NCT04225039 | Phase II in patients with recurrent glioblastoma. | Recruiting. |
| Spartalizumab | PD-1 | Stereotactic radiosurgery and  Anti-Tim-3 | NCT03961971 | Phase I in patients with recurrent glioblastoma. | Recruiting. |
| Pembrolizumab | PD-1 | CAR T-cells | NCT03726515 | Phase I in patients with newly diagnosed glioblastoma. | Active, not recruiting. |
| Nivolumab | PD-1 | Hypofractionated re-irradiation and Bevacizumab | NCT03743662 | Phase II in patients with recurrent MGMT methylated glioblastoma. | Recruiting. |
| Pembrolizumab | PD-1 | - | NCT02852655 | Phase I in patients with surgically accessible recurrent/progressive glioblastoma. | Recruiting. |
| Nivolumab | PD-1 | Ipilimumab (Anti-CTLA-4) | NCT04606316 | Phase I in patients with surgically accessible glioblastoma. | Not yet recruiting. |
| Nivolumab | PD-1 | BMS-936558 (Anti-LAG-3) and Urelumab (anti-CD137) | NCT02658981 | Phase I in patients with recurrent glioblastoma. | Active, not recruiting. |
| Nivolumab | PD-1 | Ipilimumab (Anti-CTLA-4) | NCT03233152 | Phase I following the resection in patients with recurrent glioblastoma. | Recruiting. |
| Pembrolizumab | PD-1 | NeoVax (personalized neoantigen cancer vaccine) and Standard radiotherapy | NCT02287428 | Phase I in patients with newly diagnosed glioblastoma. | Recruiting. |
| Nivolumab | PD-1 | Varlilumab (anti-CD27) | NCT02335918 | Phase I/II in patients with advanced refractory solid tumors. | Completed. |
| Pembrolizumab | PD-1 | BCA101 (EGFR/TGFβ fusion monoclonal antibody) | NCT04429542 | Phase I in patients with EGFR-driven advanced solid tumors. | Recruiting. |
| Pembrolizumab | PD-1 | Lenvatinib | NCT03797326 | Phase II in patients with selected solid tumors. | Recruiting. |
| Nivolumab | PD-1 | Bevacizumab | NCT03890952 | Phase II in patients with recurrent glioblastoma. | Recruiting. |
| Nivolumab | PD-1 | CAR T-cells and Ipilimumab (Anti-CTLA-4) | NCT04003649 | Phase I in patients with resectable recurrent glioblastoma. | Recruiting. |
| Nivolumab | PD-1 | Standard radiotherapy and Ipilimumab (Anti-CTLA-4) | NCT04396860 | Phase II/III in patients with newly diagnosed MGMT unmethylated glioblastoma. | Recruiting. |
| Pembrolizumab | PD-1 | Standard radiochemotherapy | NCT02530502 | Phase I in patients with newly diagnosed glioblastoma. | Terminated. |
| Pembrolizumab | PD-1 | TTAC-0001 (Anti- VEGFR-2) | NCT03722342 | Phase I in patients with recurrent glioblastoma. | Active, not recruiting. |
| Avelumab | PD-L1 | Hypofractionated radiation therapy | NCT02968940 | Phase II in patients with transformed IDH mutant glioblastoma. | Completed. |
| Durvalumab | PD-L1 | Hypofractionated stereotactic radiation therapy | NCT02866747 | Phase I/II in patients with recurrent glioblastoma. | Recruiting. |
| Avelumab | PD-L1 | VEGFR-2 DNA vaccine | NCT03750071 | Phase I/II in patients with progressive glioblastoma following standard treatment, with or without second surgery. | Recruiting. |
| Durvalumab | PD-L1 | Tremelimumab (anti-CTLA-4) | NCT02794883 | Phase II in patients with recurrent malignant glioma. | Active, not recruiting. |
| Durvalumab | PD-L1 | Standard radiotherapy and Bevacizumab | NCT02336165 | Phase II in patients with glioblastoma. | Active, not recruiting. |
| Avelumab | PD-L1 | - | NCT03047473 | Phase II in patients with newly diagnosed glioblastoma. | Active, not recruiting. |

Abbreviations: CAR, chimeric antigen receptor; CD137 or tumor necrosis factor receptor superfamily member 9, cluster of differentiation 137; CD27, cluster of differentiation 27; CSF1, colony stimulating factor 1; CTLA-4, cytotoxic T-lymphocyte antigen 4; DC, dendritic cell; EGFR, epidermal growth factor receptor; GITR, glucocorticoid-induced tumor necrosis factor receptor-related protein; IDH, isocitrate dehydrogenase; LAG-3, lymphocyte-activation gene 3; MGMT, O-6-methylguanine-DNA methyltransferase; PD-1, programmed cell death 1; PD-L1, programmed cell death ligand 1; TGFβ, Transforming growth factor beta; Tim-3, T cell immunoglobulin and mucin domain 3; VEGFR-2, vascular endothelial growth factor 2.
